# EMBL’s European Bioinformatics Institute (EMBL-EBI) in 2025

**DOI:** 10.1093/nar/gkaf1078

**Published:** 2025-11-13

**Authors:** Matthew Thakur, Nicolas Bosc, Cath Brooksbank, Christina Ernst, Mallory A Freeberg, Kim T Gurwitz, Henning Hermjakob, David G Hulcoop, Maria J Martin, Ellen M McDonagh, Aziz Mithani, Noel M O’Boyle, David Ochoa, Thomas Payne, Yasset Perez-Riverol, Ugis Sarkans, Alexey Sokolov, Nicole Staudt, James D Stephenson, Eleni Tzampatzopoulou, Juan Antonio Vizcaíno, Barbara Zdrazil, Johanna McEntyre

**Affiliations:** European Molecular Biology Laboratory, European Bioinformatics Institute (EMBL-EBI), Wellcome Genome Campus, Hinxton, CB10 1SD, UK; European Molecular Biology Laboratory, European Bioinformatics Institute (EMBL-EBI), Wellcome Genome Campus, Hinxton, CB10 1SD, UK; European Molecular Biology Laboratory, European Bioinformatics Institute (EMBL-EBI), Wellcome Genome Campus, Hinxton, CB10 1SD, UK; European Molecular Biology Laboratory, European Bioinformatics Institute (EMBL-EBI), Wellcome Genome Campus, Hinxton, CB10 1SD, UK; European Molecular Biology Laboratory, European Bioinformatics Institute (EMBL-EBI), Wellcome Genome Campus, Hinxton, CB10 1SD, UK; European Molecular Biology Laboratory, European Bioinformatics Institute (EMBL-EBI), Wellcome Genome Campus, Hinxton, CB10 1SD, UK; European Molecular Biology Laboratory, European Bioinformatics Institute (EMBL-EBI), Wellcome Genome Campus, Hinxton, CB10 1SD, UK; European Molecular Biology Laboratory, European Bioinformatics Institute (EMBL-EBI), Wellcome Genome Campus, Hinxton, CB10 1SD, UK; Open Targets, Wellcome Genome Campus, Hinxton, CB10 1SD, UK; European Molecular Biology Laboratory, European Bioinformatics Institute (EMBL-EBI), Wellcome Genome Campus, Hinxton, CB10 1SD, UK; European Molecular Biology Laboratory, European Bioinformatics Institute (EMBL-EBI), Wellcome Genome Campus, Hinxton, CB10 1SD, UK; Open Targets, Wellcome Genome Campus, Hinxton, CB10 1SD, UK; Wellcome Sanger Institute, Wellcome Genome Campus, Hinxton, CB10 1SD, UK; European Molecular Biology Laboratory, European Bioinformatics Institute (EMBL-EBI), Wellcome Genome Campus, Hinxton, CB10 1SD, UK; European Molecular Biology Laboratory, European Bioinformatics Institute (EMBL-EBI), Wellcome Genome Campus, Hinxton, CB10 1SD, UK; European Molecular Biology Laboratory, European Bioinformatics Institute (EMBL-EBI), Wellcome Genome Campus, Hinxton, CB10 1SD, UK; Open Targets, Wellcome Genome Campus, Hinxton, CB10 1SD, UK; European Molecular Biology Laboratory, European Bioinformatics Institute (EMBL-EBI), Wellcome Genome Campus, Hinxton, CB10 1SD, UK; European Molecular Biology Laboratory, European Bioinformatics Institute (EMBL-EBI), Wellcome Genome Campus, Hinxton, CB10 1SD, UK; European Molecular Biology Laboratory, European Bioinformatics Institute (EMBL-EBI), Wellcome Genome Campus, Hinxton, CB10 1SD, UK; European Molecular Biology Laboratory, European Bioinformatics Institute (EMBL-EBI), Wellcome Genome Campus, Hinxton, CB10 1SD, UK; European Molecular Biology Laboratory, European Bioinformatics Institute (EMBL-EBI), Wellcome Genome Campus, Hinxton, CB10 1SD, UK; European Molecular Biology Laboratory, European Bioinformatics Institute (EMBL-EBI), Wellcome Genome Campus, Hinxton, CB10 1SD, UK; European Molecular Biology Laboratory, European Bioinformatics Institute (EMBL-EBI), Wellcome Genome Campus, Hinxton, CB10 1SD, UK; European Molecular Biology Laboratory, European Bioinformatics Institute (EMBL-EBI), Wellcome Genome Campus, Hinxton, CB10 1SD, UK; European Molecular Biology Laboratory, European Bioinformatics Institute (EMBL-EBI), Wellcome Genome Campus, Hinxton, CB10 1SD, UK; European Molecular Biology Laboratory, European Bioinformatics Institute (EMBL-EBI), Wellcome Genome Campus, Hinxton, CB10 1SD, UK

## Abstract

The European Molecular Biology Laboratory’s European Bioinformatics Institute (EMBL-EBI) is one of the world’s leading sources of public biomolecular data. Based at the Wellcome Genome Campus in Hinxton, UK, EMBL-EBI is one of six sites of the European Molecular Biology Laboratory, Europe’s only intergovernmental life sciences organization. This overview summarizes the latest developments in services that EMBL-EBI data resources provide to scientific communities globally. All of the data resources described are freely available to access and reuse at https://www.ebi.ac.uk/services.

## Graphical Abstract

**Graphical Abstract:**
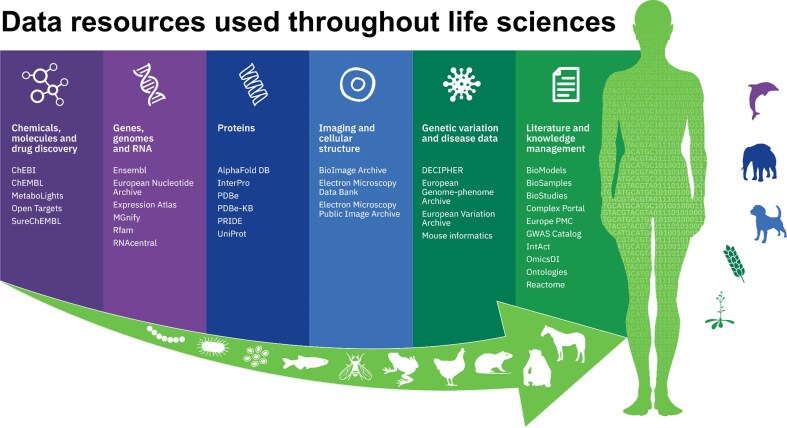

## Introduction

The European Molecular Biology Laboratory’s European Bioinformatics Institute (EMBL-EBI) is one of the world’s leading sources of public biomolecular data. Based at the Wellcome Genome Campus in Hinxton, UK, EMBL-EBI is one of six sites of the European Molecular Biology Laboratory’ (EMBL), Europe’s only intergovernmental life sciences organization. EMBL-EBI’s vision is to benefit humankind by advancing scientific discovery and impact through bioinformatics. To achieve this, EMBL-EBI collaborates with scientists, clinicians, and engineers all over the world to provide the infrastructure and tools necessary to share life science data openly.

This overview focuses on services that EMBL-EBI data resources provide to scientific communities globally and associated training activities. Companion articles in this issue provide detailed updates on the following EMBL-EBI data resources: BioSamples [1], ChEBI [2], European Nucleotide Archive (ENA) [3], Ensembl [4], Expression Atlas [5], ProteomeXchange [6], Reactome [7], and RNAcentral [8]. Here, we complement these articles by summarizing developments to the data resources not described elsewhere. EMBL-EBI data resources accessed via the EMBL-EBI services web portal comprise of the following:

Deposition databases, which archive experimental data.
Added-value databases, which provide annotation, curation, reanalysis, and integration of deposited data.
Open source software tools that enable reuse of these resources.

All EMBL-EBI data resources and many software systems can be downloaded and installed locally, and our licensing strategy is to make resources available on an open and free basis for reuse wherever possible with ‘no additional restriction on the use of the contributed data than those specified by the data owner’. EMBL-EBI data services offer bulk and machine-readable access including via API, FTP, Google BigQuery, Aspera, and Globus services.

EMBL-EBI data resources serve as foundations for hundreds of downstream external resources, research programmes, and tools. Increasingly, these include Large Language Models (LLMs) and other AI systems.

### The impact of EMBL-EBI data resources

EMBL-EBI monitors the usage and reach of the data resources it manages with its collaborators through a range of indicators, including data deposition volumes and the number of unique IP addresses that access EMBL-EBI resources and services. While each metric has its own limitations, when taken together they provide a robust picture of scale, growth, and global engagement.

**Figure 1.**
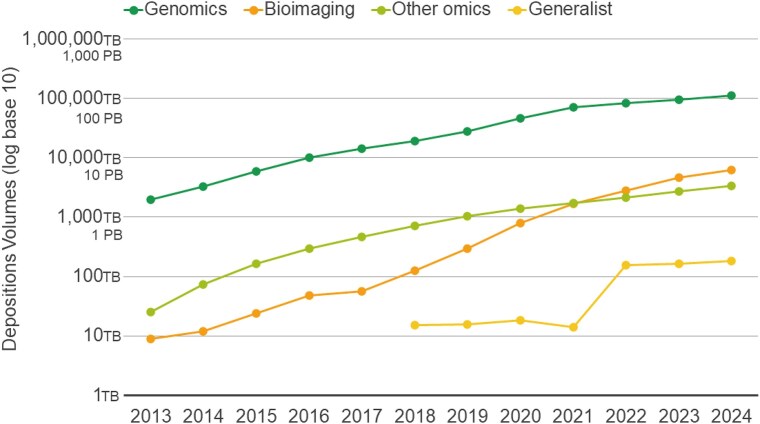
Cumulative volumes of data deposited into EMBL-EBI archival resources in TeraBytes (log scale, base 10). Genomics includes ENA, EGA, and EVA. Bioimaging includes EMPIAR, BIA, and EMDB. Other Omics include PRIDE and MetaboLights. Generalist includes BioStudies.

The rate of data submissions into EMBL-EBI’s archival resources continues to accelerate. In 2024 alone, users deposited over 16 petabytes of data, bringing the cumulative volume of deposited data to ~123 petabytes (Fig. 1). The genomics repositories ENA and European Genome–phenome Archive (EGA) [9] remain the largest archival repositories, accounting for over 90% of the total deposited volume. Notably, imaging resources are showing rapid growth in recent years reflecting the increasing demand for structured storage and access to large-scale imaging datasets via BioImage Archive (BIA) [10], Electron Microscopy Public Image Archive (EMPIAR) [11], and Electron Microscopy Databank (EMDB) [12].

**Figure 2.**
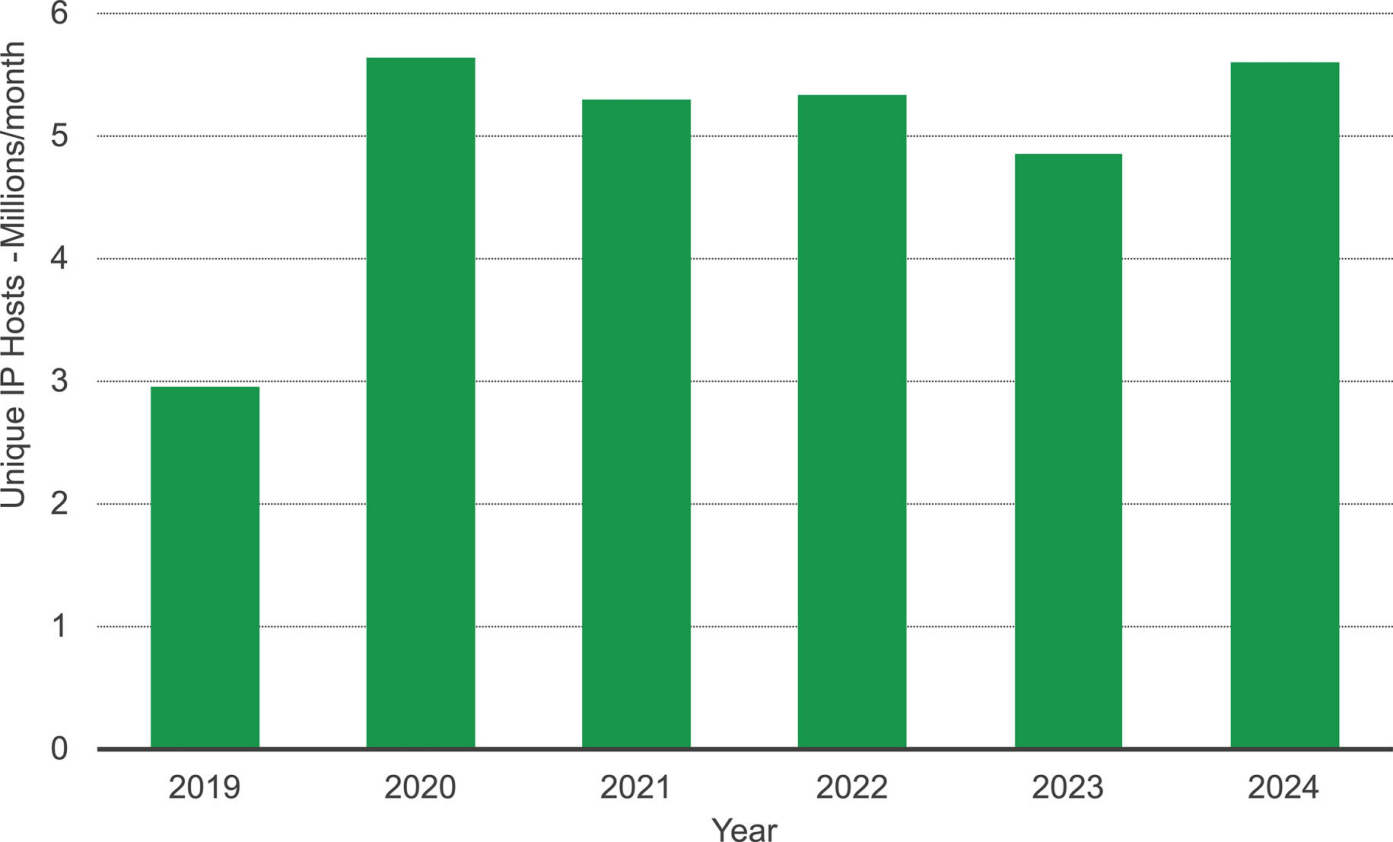
Monthly average of unique IP addresses accessing EMBL-EBI data resources from 2019 to 2024.

Following the surge in online usage during the COVID-19 pandemic, demand has remained consistently high. In 2024, an average of 5.6 million unique IP addresses accessed EMBL-EBI resources each month (Fig. 2), generating 3.5 billion web requests. EMBL-EBI services continue to support the global life science community, with every UN member state represented in the traffic data. Increasingly, users access resources via third party LLM interfaces, with traffic volume from chatbot interfaces growing exponentially and many data resources piloting Model Context Protocol servers.

## Major changes in the EMBL-EBI data resource portfolio

### A step-change in common disease genetics in the Open Targets Platform

Drugs that target gene–disease associations supported by genome wide association study (GWAS) evidence are twice as likely to succeed [13]. In 2018, Open Targets Genetics was established to help address the systematic interpretation of GWAS signals and translate them into potential new targets [14]. Due to its continuous success, the Open Targets Genetics functionalities have been fully integrated into the flagship Open Targets Platform, providing a one-stop shop for all data and analysis informing target identification [15]. Some of the new features include fine mapped credible sets derived from the GWAS catalog [16], eQTL catalogue [17], FinnGen [18], and UK Biobank Pharma Proteomics Project [19], colocalization analysis between all GWAS–GWAS and GWAS–molQTL pairs, and machine learning prediction of likely causal genes using our Locus2Gene model [20]. This refreshed interpretation of common disease variation is powered by an open-source portable pipeline named Gentropy, designed to democratize large-scale post-GWAS analysis and produce results fully compatible with the Open Targets Platform.

The new stream of target–disease evidence enhances the interpretation of disease causality when combined with 20 regularly updated data sources, 13 of which are contributed by EMBL-EBI or Open Targets. Such data feeds are designed to ensure interoperability across EMBL-EBI resources and the Open Targets Platform, as exemplified by the most recently updated NLP pipeline, OTAR Lit [21]. The newly introduced pages, such as genetic variants, studies, and credible sets, provide a new entry point to the Open Targets Platform, offering a consolidated view of information that was previously scattered. For example, the 25.06 release contained 6.5 million genetic variant pages with at least one trait or disease association derived from GWAS/molQTL credible set analysis, clinical disease annotation (ClinVar [22–24], Uniprot [25]), or pharmacogenetics (ClinPGx) [26]. All variants are extensively annotated using enhanced visualizations providing population allelic frequencies, variant effect predictions, and structural interpretation, many derived from EMBL-EBI resources such as AlphaFoldDB [27], Ensembl VEP [28], and ProtVar [29], publicly available resources such as GnomAD [30] or AlphaMissense [31], or locally run predictions on available methods such as FoldX [32].

The Open Targets Platform expands the target–disease evidence with additional prioritization factors derived from EMBL-EBI and other public resources, informing about clinical precedence, tractability, progressivity, and target safety [33]. An enhanced interface powers its prioritization capabilities allowing for on-the-fly weightings, advanced filtering, and expansion of evidence based on molecular interactions and pathways [34]. Integration of EMBL-EBI data resources into the Open Targets Platform maximizes their translational potential building therapeutic hypotheses for drug discovery.

### OPSIN, for interpretation of systematic IUPAC chemical names

The Open Parser for Systematic IUPAC Nomenclature (OPSIN) [35] is a free open-source tool that turns chemical names into molecular structures. For example, OPSIN can take ‘1,3,7-trimethylpurine-2,6-dione’, the IUPAC name for caffeine, and decode it into a MOL file, SMILES string, or InChI. Originally developed and hosted by the University of Cambridge, OPSIN has become a critical tool for translating chemical names into structured chemical representations. With over 14 000 unique visitors per month to the website, it has become widely used by researchers around the world. This website is now hosted by the Chemical Biology Services Team at EMBL-EBI in collaboration with the original developer, Dr Daniel Lowe. The migration of OPSIN to EMBL-EBI ensures the long-term sustainability of the tool and supports better integration of OPSIN with EMBL-EBI’s other Chemical Biology Services, such as integration with UniChem [36] so that interpreted chemical structures can be cross-linked to many other chemical biology databases both within the EMBL-EBI and externally.

### Perturbation Catalogue launched

Genetic perturbations influence a wide range of biological outcomes, from drug resistance to disease prognosis, but their complex mechanisms present significant data analysis challenges. Despite the abundance of data from gene, variant, and expression perturbation studies spanning diverse and often incompatible sources, this wealth of information remains under-utilized and difficult to access. Recent efforts, such as the Cancer Dependency Map for genome-wide Clustered Regularly Interspaced Short Palindromic Repeat (CRISPR) screen data in over 1000 cancer cell lines [37] and MaveDB for Multiplexed Assays of Variant Effect (MAVE) data [38], have demonstrated the value of large, harmonized datasets for improving predictive analyses.

Recognizing a need from the community, primarily through discussions with Open Targets, to bring perturbation datasets together in a unified resource, we recently launched the Perturbation Catalogue. Initially funded as part of the Open Targets research programme, the Perturbation Catalogue has been developed as a harmonized and curated resource integrating human gene (e.g. CRISPR), variant (e.g. MAVE), and expression (e.g. Perturb-seq) perturbation data. Providing both programmatic access and an interactive portal, the Perturbation Catalogue enables users to explore, visualize, and combine datasets across a wide range of biological and disease contexts. By integrating metadata and cross-references from other EMBL-EBI resources including UniProt, Ensembl, and ChEBI, the catalogue supports complex queries and facilitates the identification of shared targets and pathways across models.

The Perturbation Catalogue delivers high-quality, machine learning-ready datasets with normalized scores across perturbation types, supporting robust analyses of gene function and variant effects. Meta-analyses across individual and combined data types are also supported so that researchers can derive deeper insights across biological levels, improving our capacity to prioritize targets and interpret causal variants. The catalogue enables advanced querying and provides data visualization tools that allow researchers to rapidly test hypotheses, compare gene effects across studies, and explore functional relationships across diseases, pathways, and perturbation types. Additionally, the release of expertly curated and harmonized datasets facilitates the development of predictive models for target discovery, such as synthetic lethality in cancer or pathway-specific vulnerabilities.

**Figure 3.**
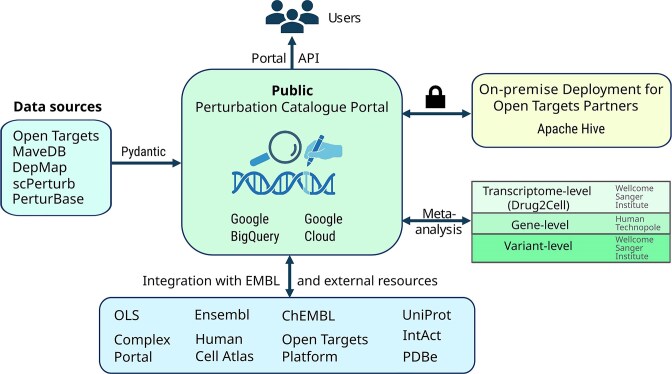
overview of the Perturbation Catalogue architecture.

The Perturbation Catalogue is accessible via a public cloud-based platform with distributed data warehousing technology. An on-premises deployable version of the catalogue is being developed to support integration of pre-publication data. The Perturbation Catalogue data portal integrates a modern, cloud-native infrastructure to enable scalable access and analysis of perturbation datasets. Metadata is indexed in Elasticsearch to provide rapid and flexible search, while Google BigQuery serves as the central data warehouse and single source of truth. The web platform is implemented using FastAPI with Pydantic models for robust data validation, and interactive data exploration and visualization are delivered through Python Dash (see architecture overview in Fig. 3).

Future work will focus on leveraging the Perturbation Catalogue to enable integrated meta-analyses across gene, variant, and expression perturbation data, ultimately building multi-layered associations across biological levels. This work will support the identification of disease-relevant gene networks, prioritization of targetable variants, and deeper insights into phenotype–genotype relationships, particularly in complex disease areas such as cancer, immunology, and neurodegeneration.

## New features and applications of existing data resources

### IntAct CytoScape integration

The web interface of the IntAct database [39] of molecular interactions provides comprehensive search capabilities, but is for performance reasons limited to visualizing a maximum of 1500 interactions. In July 2025, we released a new version of the IntAct Cytoscape app [40] that supports a smooth transition between the web-based IntAct interface and the Cytoscape app when a search results in >1500 interactions. The new app also allows interaction network merging based on Panther [41] protein orthology.

### Enhanced data provenance in ChEMBL and new antiviral bioactivity data

With release 35 of the ChEMBL database of bioactivity data [42], new database fields have been introduced to further enhance the provenance and FAIRness of the bioactivity data hosted in ChEMBL. A new CONTACT field has been added to the DOCS table, designed to provide a stable identifier (ideally an ORCID) for one to three primary contacts associated with a dataset. These individuals may be the experimenters, laboratory heads, or line managers responsible for the work.

To capture more detailed information about the origin of deposited datasets, two new fields—SRC_COMMENT and SRC_URL—have been added to the SOURCE table. SRC_COMMENT offers a concise summary of the data source, including the nature of the data provided and the institution or company that supplied it to ChEMBL, while SRC_URL provides a direct link to the depositor’s website with relevant information about the source. In addition, the SOURCE_DESCRIPTION field has been harmonized to follow a more consistent naming convention, offering clearer descriptions of the data type and its origin (e.g. institute, company, or research group).

A new ASSAY_GROUP field has also been introduced to the ASSAYS table. This groups assays from different depositions that use exactly the same assay setup, enabling users to identify comparable assays as defined by depositors.

The Chemical Biology Services team continues to collaborate closely with both new and existing data depositors to accommodate emerging data types. A recent example is the deposition of a large collection of assays and bioactivity data for antiviral targets—including SARS-CoV-2, Zika virus, and Dengue virus—by the AI-driven Structure-enabled Antiviral Platform (ASAP) consortium. Funded by the US National Institute of Allergy and Infectious Diseases, ASAP is dedicated to developing novel chemical assets with antiviral activity against viral families with future pandemic potential. The consortium applies a state-of-the-art, structure-enabled discovery paradigm, leveraging advances in AI/ML and computational chemistry to identify, prioritize, and prosecute discovery campaigns for new antiviral agents.

### Refactoring of SureChEMBL and expanded patent coverage

The Chemical Biology Services team has also released a major update to their annotated patent database: SureChEMBL2.0 [43]. A member of the EMBL-EBI database family for over a decade, SureChEMBL has recently undergone substantial infrastructure and user interface refactoring, laying the foundation for enhanced functionality and usability. Patent coverage has been expanded to include documents from the China National Intellectual Property Administration, in addition to those from the US Patent and Trademark Office, the World Intellectual Property Organization, the European Patent Office, and the Japan Patent Office. The database now comprises over 43 million patent documents and >31 million extracted chemical structures. The chemical structure extraction pipeline has also been made more open and transparent by adopting the same compound standardizer as ChEMBL, and by integrating the open-source cheminformatics toolkit RDKit at multiple stages of compound registration. To support large-scale downstream analysis, data accessibility has been improved through the introduction of bulk downloads available from our FTP site as bi-weekly dumps of SureChEMBL content that include compound–patent relationships.

### Developments in the resources for proteomics and metabolomics data: PRIDE and MetaboLights

The PRIDE database continues to be the world-leading resource for proteomics data deposition, within the ProteomeXchange consortium. As of July 2025, PRIDE had archived a total of 50 804 proteomics datasets. Notably, between July 2024 and July 2025, the number of submitted datasets increased by 23% (1557 datasets in total), when compared to the previous 12 months (July 2023–July 2024). One of the reasons behind this growth is the introduction of the Globus file-transfer protocol for large-scale data submissions. We have also further facilitated data downloads to make the reuse of public proteomics data easier, through pridepy [44], a Python client that streamlines programmatic access to PRIDE public and private datasets, supporting multiple file transfer protocols (FTP, Aspera, Globus). Aspera provides the most consistent performance across global locations. The client’s REST API integration enables automated workflows for dataset searching, metadata access, and large-scale data retrieval, addressing critical needs in cloud-based and HPC environments.

PRIDE is also expanding the use of LLMs, building on the PRIDE Chatbot [45], released in 2024. For example, the team currently employs the Gemini 2.5 model in combination with EuropePMC data to automatically annotate datasets in PRIDE that are part of multiomics studies. Overall, >8000 PRIDE datasets have been systematically linked to the corresponding omics datasets coming from the same studies in other resources such as GEO (transcriptomics datasets), MetaboLights (metabolomics), and ENA and EGA (genomics), among many others. This represents a breakthrough in establishing multiomics data connections across diverse global resources. An illustrative case is PRIDE dataset PXD063552, which was annotated as part of a multiomics study using this workflow. Additionally, the PRIDE Archive USI (Universal Spectrum Identifier) resource has been further developed, enabling the access and visualization of every mass spectrum stored in PRIDE. Beyond the originally submitted mass spectra data, PRIDE Archive USI also supports alternative interpretations of the peptide spectrum matches, including peptides with their corresponding post-translational modifications (PTMs), amino acid variants, and alternative peptide sequences. Widely used proteomics data-based resources such as Scop3P [46] and MatrisomeDB [47] are now integrated with PRIDE Archive USI, allowing their users to directly explore their peptide evidence alongside the supporting mass spectra.

In the context of data reuse and dissemination of public proteomics data, the team continues to maximize the value of public datasets [48] by enabling the development of new algorithms [49] for large-scale data reanalysis, built on the SDRF-Proteomics metadata standard [50] and the quantms workflow [51]. Reanalysed datasets generated by the team and close collaborators continue to be systematically integrated into other EMBL-EBI resources, including UniProt and Expression Atlas. In 2025, the PRIDE reanalysis activities expanded beyond baseline protein expression and PTM data: Pilot studies were performed to integrate metaproteomics data in MGnify [52] and to integrate single amino acid variants coming from RNA editing events into the resource REDIportal [53]. Integration of quantitative proteomics data in the Open Targets platform is work in progress.

MetaboLights is one of the leading databases for the deposition of studies in metabolomics and related fields such as lipidomics and exposomics. MetaboLights has continued to experience a very significant growth in the number and diversity of submitted studies: a record number of 438 complete studies were accessioned during the first 6 months in 2025 (with a total number of 1467 ongoing). As of August 2025, data hosted in MetaboLights (>300 TB in total) comprise 7397 different organism/organism parts, with user accounts from across 146 countries. This growth however presents challenges and the MetaboLights team spent much of the last year evolving the resource with a new simplified submission workflow, which reduces reliance on manual curation and assigns permanent identifiers (MTBLSxxx) only after submitted datasets are complete and validated. Other recent developments include the new study validation framework (based on Open Policy Agent) and the implementation of automated metadata updates (fixes), a new revision mechanism for public studies, open-source libraries (metabolights-utils on PyPI), and updated documentation (MkDocs). These developments enabled applications to be updated, such as the MetaboLights Online Editor, as well as separated in a microservice-based architecture with a complete migration of the infrastructure to Kubernetes.

As a key point, in 2025, MetaboLights started to coordinate the new MetabolomicsHub consortium, aiming to bring the main metabolomics data resources together with the aim to standardize open data practices within the metabolomics field worldwide—inspired by analogous projects in other fields such as ProteomeXchange for proteomics and following recently initiated efforts for data harmonization in metabolomics data repositories [54]. In addition to MetaboLights, MetabolomicsHub has the participation of the US-based resources Metabolomics Workbench [55] and GNPS/MassIVE [56].

### Functional genomics submission via Annotare

Annotare [57] is EMBL-EBI’s web-based submission tool for functional genomics data archived in the ArrayExpress collection within BioStudies [58]. It provides an interactive interface guiding researchers through the preparation of metadata and file upload, ensuring compliance with community standards such as MINSEQE and minSCe [59]. While the majority of submissions are bulk and single-cell RNA-seq, Annotare also supports a wide range of assay types, such as chromatin accessibility, epigenetic modifications, RNA–protein interactions, and chromatin conformation, among others. This breadth is essential for accommodating the increasingly multi-modal experimental designs in functional genomics.

With most datasets being sequencing based, Annotare acts as an internal broker, routing raw sequencing files to the ENA, while the ArrayExpress collection in BioStudies serves as a metadata hub. This enables the capture of sample- or technology-specific information not represented in ENA—which is particularly critical for single-cell experiments—as well as the deposition of derived data files such as count matrices for bulk and single-cell transcriptomic datasets, genome browser tracks, or peak files from immunoprecipitation assays. No restrictions are placed on file size or type for these derived outputs, and their inclusion is strongly encouraged to maximize dataset reusability.

Annotare usage is steadily increasing. In 2024, 1639 new submissions were initiated and 1102 completed within the same year. This upward trend has accelerated in 2025, with 1367 new submissions recorded between January and August alone—25% more than in the same period of 2024. To meet the demands of ever-larger datasets, Annotare recently introduced Globus as an option for data transfer, offering faster and more stable uploads alongside existing methods.

### Updates to AntiFam—a collection of spurious protein families

AntiFam is a collection of profile hidden Markov models (profile-HMMs) designed to identify spurious protein predictions in sequence databases and metagenomic projects. The resource contains models derived from two main sources: previously identified erroneous gene predictions including Shadow ORFs and their homologues, and translations of commonly occurring non-coding RNAs such as transfer RNAs. Release 8.0 of AntiFam contains 278 profile-HMM families, expanding from 263 families in the previous release. The database provides both a comprehensive HMM library and superkingdom-specific sets for Eukaryota, Bacteria, Archaea, and Viruses, allowing targeted quality control across different taxonomic groups. Notably, validation using AlphaFold structure prediction led to the identification and removal of one AntiFam entry (ANF00096) that was found to represent a bona fide protein family with predicted globular structure, demonstrating the value of structural analysis for quality control of the resource. AntiFam serves as an essential tool for UniProt and other protein sequence databases, helping to filter out spurious open reading frames that may arise from incorrect gene predictions or translations of non-coding sequences. AntiFam matches are now accessible through the InterPro website, increasing visibility and accessibility of spurious protein annotations. The resource is freely available under the Creative Commons Zero licence, with data downloadable from https://ftp.ebi.ac.uk/pub/databases/Pfam/AntiFam/, and can be searched using HMMER3 [60] software with gathering threshold cutoffs.

### Enhancements of annotations, predictions, and visualizations to further facilitate interpretation of protein coding variation in ProtVar

ProtVar, the EMBL-EBI tool to contextualize and evaluate human missense variation in proteins, has undergone substantial improvements since launching in 2023 [61]. Users can now retrieve annotations for their variants using complementary DNA and protein position in addition to genomic coordinates and a broader range of variant identifiers. A new feature allows the browsing of all known variants and annotations for any human protein, including any possible missense variants not yet reported in databases. The tool now includes tens of millions of new variants from GnomAD [30], ClinVar [23], and COSMIC [62] as well as newly added GnomAD allele frequencies now covering over 93% of the human proteome.

**Figure 4.**
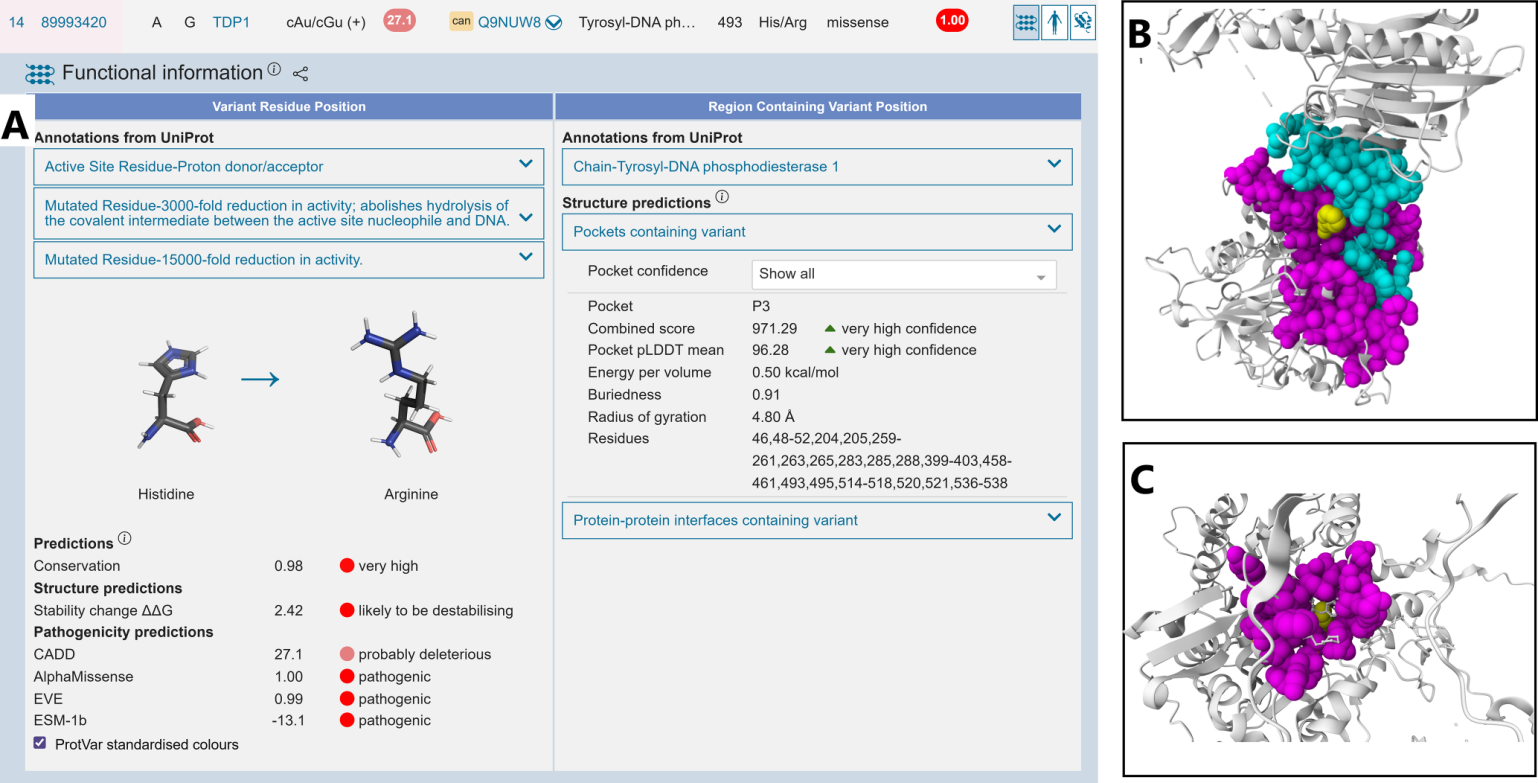
ProtVar outputs for three different missense variants: (**A**) functional annotations results page for a single missense change including curated annotations and predictions, (**B**) variant (yellow) at the interface between two proteins, interface residues shown in cyan and magenta, and (**C**) variant in a predicted pocket showing the relative position of ligands.

In addition to adding new variant predictors such as Alphamissense [31] and ESM-1b [63] (Fig. 4A), the number of precalculated missense changes calculated using FoldX [64] has increased from 6M to 208M, covering the majority of the structurally defined proteome. Protein visualizations have also been enhanced in order to highlight variants in the context of the continually increasing number of predicted protein–protein interfaces (Fig. 4B) and protein pocket (Fig. 4C) locations. AlphaFill [65] models have also been incorporated into the Mol* [66] pocket visualizations to highlight the relative position of experimentally derived ligands on AlphaFold structures (Fig. 4C). With new linking, job history retrieval and sharing facilities recently developed, in addition to new API endpoints, ProtVar continues to facilitate industry, academia, and clinical geneticists to interpret and contextualize human missense variation.

### Pilot of the BioAIrepo AI model repository

Researchers from across the life sciences increasingly build and use machine learning models for gaining better understanding of biological processes. Approaches to sharing and publishing this work are still developing. If models are shared at all, generic AI model platforms (such as huggingface.co) or code repositories are used. For selected types of data specialized ‘model zoos’ exist, such as bioimage.io for imaging. BioAIrepo is a new collection within EMBL-EBI’s BioStudies database that offers means for scientists to share their AI work by describing what their models do, how they have been constructed, and, critically, providing links to datasets used for model building, testing, and validation. As of September 2025, it contains a small set of models across a variety of domains: microscopy, splicing prediction, protein structure determination, and analysis of omics data analysis. This work will be continued, establishing a more structured approach to document models across disciplines, providing better means to find and reuse this information and enabling scientists to submit their models and associated data links in a streamlined manner, while also encouraging adherence to the DOME guidelines (dome-ml.org/).

## Training

EMBL-EBI’s training programme continues to equip scientists and service-delivery staff with the skills to make effective use of bioinformatics resources while promoting best practice in open and FAIR data management, combining live and on-demand training with community partnerships to expand the reach and impact of our activities. Each year, around 500 scientists attend our live courses, offered either in person or virtually, while ~500 000 annual unique IP users access our freely-available, web-based, on-demand content. We also engage in bioinformatics trainer communities, providing support for and learning from other trainers, both within EMBL and externally, helping to strengthen capacity across the scientific community and staying at the cutting edge of training best practices.

A major 2025 highlight is the launch of the AI Training Assistant, designed to guide learners through EMBL-EBI’s extensive catalogue of training materials and to support more personalized learning experiences, alongside updated dedicated Service training pages to help users easily find all relevant training content for specific EMBL-EBI resources, including links to related training materials across the wider portfolio. Our ongoing partnership efforts are captured on the refreshed partnerships page. Alongside longstanding collaborations with ELIXIR and the ISCB Education community, the page features new initiatives such as BiotrAIn, a collaboration with CABANAnet that aims to create a fundamental and sustainable curriculum and associated community of practice on AI for bioscientists from Latin America.

Our live training portfolio continues to expand, with new EMBO-funded courses joining the programme, including ‘Methods for infectious disease modelling using genomics (2025)’ and ‘Causality in biomedicine: going beyond associations (2026)’. Financial assistance schemes have been broadened, enabling participants from low- and middle-income countries to attend EMBL-EBI courses. Alongside these developments, new on-demand tutorials and structured learning pathways have been added to our library, featuring content on emerging methodologies and LLMs.

Webinar highlights from 2025 include dedicated series on LLMs and their applications in bioinformatics, and organizing and sharing imaging data, adding to our established thematic collections. A notable community event was the AlphaFold Education Summit, co-organized with Google DeepMind, which brought together experts and learners to explore cutting-edge uses of AlphaFold in research and teaching in LMICs.

Our communication channels continue to evolve, with training activities promoted across an established LinkedIn presence, which now has over 35 000 followers, and a growing presence on newer platforms such as BlueSky. This supports our goal of broadening awareness and access to EMBL-EBI’s training opportunities and ensuring that learners worldwide can benefit from our expertise.

## Conclusion

The transformative rise of artificial intelligence across the life sciences has created unprecedented demand for high-quality, well-annotated datasets that can serve as foundations for robust AI applications. Meeting this demand requires a resilient global data resource infrastructure that builds on the operating and funding models of longstanding resources, while exploring new models and ways of working. Biological data resources constantly evolve to meet changing scientific needs and technological opportunities, demonstrated in the developments shared above. EMBL-EBI data resources will continue to provide users with high-quality, expert-annotated open data and tools at scale, providing the foundations for transformative research insights and high impact applications of biodata.

## Data Availability

All of the data resources described above are freely available to access and reuse at https://www.ebi.ac.uk/services.
